# Augmented plasma microparticles during acute *Plasmodium vivax *infection

**DOI:** 10.1186/1475-2875-9-327

**Published:** 2010-11-16

**Authors:** Fernanda MF Campos, Bernardo S Franklin, Andréa Teixeira-Carvalho, Agnaldo LS Filho, Sálua CO de Paula, Cor J Fontes, Cristiana F Brito, Luzia H Carvalho

**Affiliations:** 1 Centro de Pesquisas René Rachou/Fundação Oswaldo Cruz, Av. Augusto de Lima 1715, Belo Horizonte, MG 30190-002, Brazil; 2 Departamento de Clínica Médica, Universidade Federal de Mato Grosso, Rua Luiz Phellipe Pereira Leite sn, Alvorada, Cuiaba, MT, 78048-902, Brazil; 3 Departamento de Ginecologia e Obstetrícia, Faculdade de Medicina, Universidade Federal de Minas Gerais, Avenida Professor Alfredo Balena 190, Belo Horizonte, MG, 30130-100, Brazil

## Abstract

**Background:**

In the last few years, the study of microparticles (MPs) - submicron vesicles released from cells upon activation or apoptosis - has gained growing interest in the field of inflammation and in infectious diseases. Their role in the human malaria parasite *Plasmodium vivax *remains unexplored. Because acute vivax malaria has been related to pro-inflammatory responses, the main hypothesis investigated in this study was that *Plasmodium vivax *infection is associated with elevated levels of circulating MPs, which may play a role during acute disease in non-immune patients.

**Methods:**

Plasma MPs were analysed among thirty-seven uncomplicated *P. vivax *infections from an area of unstable malaria transmission in the Brazilian Amazon. The MP phenotype was analysed by flow cytometry using the classical MP marker, annexin, and fluorochrome-labeled monoclonal antibodies against specific cell surface markers. The frequencies of plasma MPs in *P. vivax *patients (n = 37) were further compared to malaria-unexposed controls (n = 15) and ovarian carcinoma patients (n = 12), a known MPs-inducing disease non-related to malaria.

**Results:**

The frequencies of plasma circulating MPs were markedly increased in *P. vivax *patients, as compared to healthy age-matched malaria-unexposed controls. Although platelets, erythrocytes and leukocytes were the main cellular sources of MPs during vivax malaria, platelet derived-MPs (PMPs) increased in a linear fashion with the presence of fever at the time of blood collection (β = 0.06, p < 0.0001) and length of acute symptoms (β = 0.36, p < 0.0001). Finally, the results suggest that plasma levels of PMPs diminish as patient experience more episodes of clinical malaria (β = 0.07, p < 0.003).

**Conclusions:**

Abundant circulating MPs are present during acute *P. vivax *infection, and platelet derived-MPs may play a role on the acute inflammatory symptoms of malaria vivax.

## Background

*Plasmodium vivax *is the most widespread *Plasmodium *species being responsible for up to 390 million clinical cases each year [1,2]. For long, *P. vivax *infection was considered a benign and self-limited disease, especially when compared to the burden of *Plasmodium falciparum *infection. Recent studies from Indonesia, Papua New Guinea and India have, however, highlighted the association between vivax malaria and life-threatening manifestations, such as respiratory distress, severe anaemia, and neurological manifestations with coma [3-5], placing *P. vivax *infection in a higher status as a global health concern.

An additional neglected effect of the "benign" *P. vivax *infection concerns its poorly explored pathogenesis. While a large bulk of literature on the immunological and inflammatory patterns of falciparum malaria is available, the pathogenesis of vivax malaria was not submitted to such scrutiny. It is, nevertheless, intriguing that *P. vivax *infection triggers fever with a lower peripheral parasitaemia [6-8]. The few studies that had addressed the pathogenesis of vivax malaria had shown that *P. vivax *infection induces greater host inflammatory responses compared to *P. falciparum *malaria [9-11] with pro-inflammatory cytokines levels being closely associated with disease severity in some studies [10-13]. Hence, the different clinical presentations of vivax malaria infection may be related to the intensity of pro-inflammatory responses. In this regard, the search for sensitive and predictive inflammatory biomarkers in vivax malaria may enhance early detection of severe cases.

Cellular microparticles (MPs) - submicron (< 1 μm) membrane vesicles released from cells into the blood stream in response to a variety of stimuli, i.e. cell activation, stress, serine proteases, pro-inflammatory cytokines and apoptosis, have emerged as potential inflammatory biomarkers as elevated levels are found in plasma during many diseases of inflammatory etiology [14]. Consequently, this heterogeneous population of small, membrane-coated vesicles has been used as sensitive markers of diverse process, such as inflammation, coagulation, vascular function and apoptosis. In addition, MPs are important carriers of membrane components or bioactive molecules [15] being involved in a series of "gain of function" phenomena [16]. As an example, MPs derived from activated platelets have been shown to play a role on the pathogenesis of *P. falciparum *cerebral malaria by transferring platelet antigens to the surface of iRBCs and dramatically enhancing their cytoadherence to endothelial cells within brain micro vessels [17,18]. MPs have also been shown to contribute to inflammation during *P. berghei *malaria by inducing potent activation of macrophages [19]. However, their role in vivax malaria pathogenesis has not been investigated.

The main hypothesis to be investigated here is that *P. vivax *infection is associated with elevated levels of circulating MPs of different cellular origins, and those MPs play a role in *P. vivax *induced inflammation. For this purpose, the phenotype of circulating MPs in plasma from *P. vivax *malaria patients in a hypoendemic area of the Brazilian Amazon was assessed. As control, the plasma MP phenotype from healthy malaria-unexposed donors and patients with ovarian carcinoma, a known MPs-inducing disease non-related to malaria, was simultaneously investigated. The results showed that platelet (PMPs), erythrocyte (EMPs) and leukocyte (LMPs) derived-MPs are significantly increased in plasma from *P. vivax *patients as compared to healthy donors. Interestingly, the frequencies of annexin V/CD41a double-positive MPs (PMPs) were associated with the length of acute illness and the presence of fever at the time of blood collection. The results presented here comprise the first evidence of the presence of MPs during vivax malaria and offer a new tool for the study of the pro-inflammatory patterns of *P. vivax *infection.

## Methods

### Study site and subjects

This study was conducted between January and November 2008, in the outpatient clinic of the Tropical Medicine Center of Porto Velho (CEMETRON), the capital of Rondônia State, Brazilian Amazon area. In the Amazon area, malaria is mainly an occupational disease, with population at risk largely represented by adult males. Low rates of transmission occur throughout the year with large dominance of vivax over falciparum malaria [20]. The main vector is *Anopheles darlingi *and the number of infective bites associated with malaria is estimated to be between 2 to 10 per inhabitant per year [21].

Individuals who sought care at CEMETRON and whose thick blood smear was positive for *P. vivax*, were invited to participate in the study. Selected volunteers were all negative for *P. falciparum *and/or *Plasmodium malariae *infection by both microscopic examination and a nested-PCR, carried-out latter. Thirty-seven patients, aging 15 to 66 years, were enrolled in the study after the written informed consent, in accordance with guidelines for human research, as specified by the Brazilian National Council of Health (Resolution 196/96). Antimalarial and supportive therapies were given according to standard protocols.

Clinical and demographical data were acquired through a standardized questionnaire, and the haematological profile was assessed by automated complete blood count carried-out at CEMETRON's hematology facility. Table 1 summarizes demographic, epidemiological, parasitological and hematological data of *P. vivax *infected-volunteers.

**Table 1:** Characteristics of the thirty seven uncomplicated *Plasmodium vivax *patients

CHARACTERISTICS	
**Gender, male:female**	2.7:1
**Median Age, years (range)**	36 (15-66)
**N**° **of previous malaria episodes, n (%)**	
≤ 5	19 (51.3)
6 - 10	13 (35.1)
> 10	5 (13.5)
**N**° **of days of acute illness, n (%)**	
≤ 2	10 (26)
3 - 5	19 (51.3)
> 6	8 (21.6)
**Symptoms in the last 3 days, n (%)**	
Fever	37 (100)
Headache	33 (89.1)
Chills	35 (94.6)
Myalgia	32 (86.4)
Anorexia	24 (64.8)
Nausea	13 (35.1)
Diarrhoea	7 (18.9)
Vomiting	6 (16.2)
Dyspnea	4 (10.8)
**Parasitaemia, parasites/μl of blood, n (%)**	
≤ 500	10 (27.0)
500 - 10,000	10 (27.0)
> 10,000	17 (45.9)
**Body temperature (**^**o**^**C)**	
≤ 36	8 (21.6)
37 - 37.8	14 (37.8)
> 37.8	15 (40.5)
**Haemoglobin levels g/dL, median (range)**	13.26 (9.7 - 16.6)
**Platelet counts ×1000/mm**^**3**^**, median (range)**	115 (53 - 208)
**Leukocyte counts ×100/mm**^**3**^**, median (range)**	52 (23 - 117)
**Haematocrit %, median (range)**	41 (32.2 - 50.6)

In the studied area, subjects were migrants from malaria-free areas. Consequently, their ages do not necessarily correlate with malaria exposure. Cumulative exposure to malaria was, therefore, based on self-reported number of lifetime malaria episodes. As their previous malaria experience showed a variable number of infections, extreme values and the possibility of recall bias were avoided by categorizing patients into three groups: i) five or less previous malaria episodes (PME); ii) 6 to10 PME; and ii) more than 10 PME. Venous blood was obtained from patients on admission, and in those with fever (above 37.5°C) at the time of blood collection. In five patients it was possible to take additional samples, during convalescence, on day 7 and 21 after treatment. Plasma samples from fifteen age-matched malaria-unexposed donors were used as baseline control. As MPs positive samples plasma from 12 patients, aging 45 to 65 years, diagnosed with ovarian carcinoma, as it has been shown that cancer cells from this highly lethal gynaecological malignancy can release MPs [22].

### Purification of MPs from plasma

MPs were prepared as described elsewhere [17,19]. Briefly, Citrated blood (0.5 mL) was centrifuged at 1,500 × *g *for 15 min, and plasma was then cooled to -20°C before storage at -80°C. Samples were further centrifuged at 13,000 *x g *for 3 min to obtain platelet-free plasma. The latter was diluted 1:3 in citrated PBS containing heparin and centrifuged at 14,000 *x g *for 90 min at 15°C. The resultant MP pellet was then resuspended in 1× annexin V binding buffer (BD Biosciences, California, US).

### Flow cytometry assays

**Figure 1:**
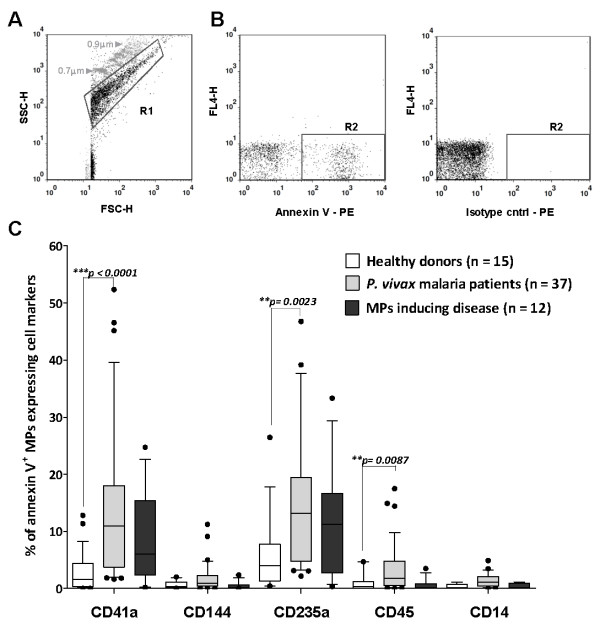
**Identification of plasma MPs**. (A) MPs isolated from plasma were gated (R1) based on their forward (FSC) and side (SSC) scatter distribution as compared to 0.7 - 0.9 μm synthetic Nile Red microparticles (grey dots). (B) Events present in R1 were accessed for their annexin V positive staining using PE-conjugated mAbs. Mouse IgG PE-conjugated isotype control mAbs were used to place gates accurately (R2). (C) FITC immunolabeling for the cell markers CD41a (platelets), CD144 (endothelial cells), CD325a (erythrocytes), CD45 (leukocytes) and CD14 (monocytes) was further assessed within annexin V^+ ^gated events (R2) and were compared in plasma samples from healthy donors, *P. vivax *malaria and ovary carcinoma patients (MPs-induced disease). Boxes represent median and interquartile interval, whiskers represent the 10 and 90 percentiles. Means were compared using the Mann-Whitney two-tailed test. A p value < 0.05 was considered significant.

Unless otherwise stated, all reagents and mAbs used in the Flow cytometry experiments were from BD Biosciences. MPs isolated from plasma were gated (R1) based on their forward (FSC) and side (SSC) scatter distribution as compared to the distribution of synthetic 0.7 - 0.9 μm SPHERO™ Amino Fluorescent Particles (Spherotech Inc. Libertyville, Illinois, US) (Figure 1A). Taking into account the presence of PS residues in MPs surface, events present in R1 were accessed for their positive staining for annexin V (BD Bioscience) - a classical marker for microparticles - using PE-conjugated monoclonal antibodies (mAbs) (Figure 1B). Mouse IgG PE conjugated isotype control mAbs were used to properly place gates. Annexin V^+ ^events gated within the R2 region were further assessed for immunolabeling with FITC-conjugated mAbs against the cell markers CD41a (platelets), CD144 (endothelial cells), CD235a (erythrocytes), CD45 (leukocytes) and CD14 (monocytes) or the correspondent mouse IgG FITC-conjugated isotype control mABs. The samples were analysed in a Flow Cytometry FACSCalibur (Becton-Dickinson, California, US). Over 100,000 events were acquired on each sample, to reach at least 2,000 events within the MPs gate.

### Quantification of plasma MPs levels

To confirm if the absolute numbers of plasma MPs were different between malaria patients and healthy donors, as well as to determinate the numbers of plasma MPs per microliter (MPs/μl), the cytometer was set to operate at a high flow rate setting for 60 seconds for each sample. The number of MPs/μl of plasma was calculated as described elsewhere [18]: MPs/μl = (N × 400)/(60 × 100), in which N is the number of events, 400 is the total volume of in the tube before analysis, 60 is the sample volume analysed, and 100 is the original volume of MPs suspension.

### Statistical analysis

**Figure 2:**
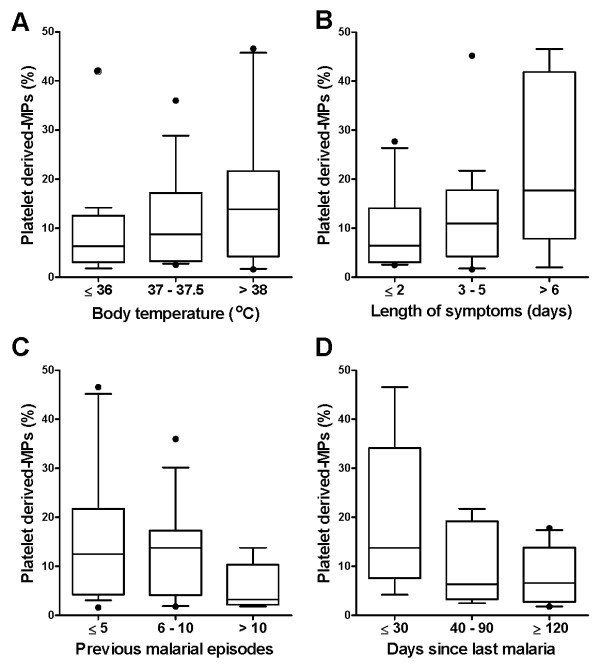
**Platelet derived MPs (PMPs) correlate with fever and length of symptoms of acute malaria and may be impacted by cumulative clinical malarial episodes**. *P. vivax *malaria patients were categorized according to their body temperature (°C) (A); prolonged symptoms (B); cumulative malarial episodes (median 5, range 0 - 60) (C); and the time since last malarial episode (D). Boxes represent median and interquartile interval, whiskers represent the 10 and 90 percentiles. Data were transformed into neperian logarithm before applied to linear regression analysis. Positive linear trends were observed between the frequency of PMPs in plasma and presence of fever (above 37.5°C) at the time of blood collection (β = 0.06, p < 0.0001), length of malaria symptoms, estimated here by number of days with acute illness (β = 0.36, *p < 0.0001*), and previous clinical malaria episodes (β = 0.07, p < 0.003).

Data were analysed using Graph pad Instat 4.0 and SPSS 15.0 statistical software. Differences in the means of the frequencies (double staining for annexin V and cell markers) between groups were analysed using two-tailed student's t test or Mann-Whitney when data did not fit a Gaussian distribution. The log-transformation of data was applied for situations where variances normality assumptions failed followed by linear regression to investigate the association between the clinical parameters and MPs levels. Pairwise correlations were evaluated with Pearson's correlation coefficient ρ. Multiple linear regression models with stepwise backward deletion were built to describe independent associations between covariates (fever, age and number of previous malaria episodes) and the presence of PMPs. A P value < 0.05 was considered to be statistically significant. For illustration purpose (as shown in Table 1 and Figure 2) the patients were categorized into sub-groups according to their body temperature, the numbers of previous malarial episodes, length of symptoms (in days), and days since last malarial episode.

## Results

### Platelet, erythrocyte and leukocytes are the main sources of plasma MPs during acute *P. vivax *malaria

The phenotype of plasma circulating MPs during acute vivax malaria was investigated, with specific mAbs for cell markers i.e. CD41a, CD235a, CD45, CD144 and CD14 were used to discriminate the cellular sources of annexin V^+ ^MPs. The percentages of annexin V^+ ^MPs stained for each cell marker was compared between samples from *P. vivax *malaria patients, healthy donors or samples from a MPs-induced disease non-related to malaria (Figure 1C).

The frequency of platelet (PMPs) (*p < 0.0001*), erythrocyte (EMPs) (*p = 0.0023*) and leukocyte derived-microparticles (LMPs) (*p = 0.0087*) were significantly increased in plasma samples from malaria patients as compared to healthy donors. The increased levels of PMPs, EMPs and LMPs were confirmed when the absolute number of MPs per μl of plasma was calculated (additional file 1). As expected, the frequencies of PMPs and EMPs, but not LMPs, were also significantly increased in plasma from patients with ovarian carcinoma (*p < 0.05*). These results suggest that platelets, erythrocytes and leukocytes are the main source of plasma circulating MPs during acute *P. vivax *malaria and its presence may be associated with inflammation.

### Plasma MPs associate with acute malaria symptoms

To investigate the role of MPs in the pathogenesis of vivax malaria, potential factors that could be associated with the presence of circulating MPs in acute *P. vivax *infection were analysed, including demographic, clinical, haematological and parasitological parameters. No association was found between levels of MPs and haematological (haemoglobin levels, haematocrit, platelet or leukocyte counts) or parasitological data. Of note, positive linear trends were observed between the presence of fever at the time of blood collection and the frequency of PMPs in plasma (β = 0.06, p < 0.0001) (Figure 2A). Significant association was also observed between higher frequencies of PMPs and prolonged malaria symptoms, estimated here by number of days with acute illness (β = 0.36, *p < 0.0001*) (Figure 2B). More specifically, patients reporting symptoms in the last four or more days before seeking care at CEMETRON had significant higher levels of PMPs in plasma when compared to patients reporting more immediate symptoms. Apparently, the levels of PMPs in plasma diminish as previous clinical malaria episodes increases (β = 0.07, p < 0.003) (Figure 2C). No significant association was found between PMP levels and days elapsed since last malarial episode (Figure 2D).

Although manifestation of acute malaria such as fever can be influenced by different factors including host age and immunological background - evaluated here by previous malaria experience - multiple linear regression models confirmed the independent association between fever and microparticles, as well as length of symptoms and microparticles (p < 0.001).

### Plasma MPs levels decrease after anti-malarial chemotherapy

**Figure 3:**
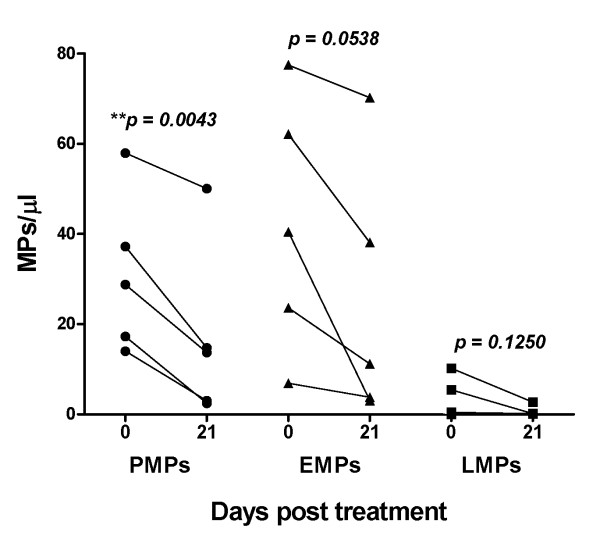
**Plasma MPs levels decrease after anti-malarial chemotherapy**. Levels of platelet (PMPs), erythrocyte (EMPs) and leukocyte derived-MPs (LMPs) were quantified in plasmas from five patients at the acute phase of *P. vivax *infection (0) and after 21 days of anti-malarial chemotherapy. MP levels were calculated and expressed as number/μl of plasma as detailed in Methods. Statistical analyses were performed using the two-tailed paired t-test. A p value < 0.05 was considered significant.

In five *P. vivax *patients, plasma MPs levels were assessed 7 and 21 days after anti-malarial chemotherapy. The levels of plasma MPs remain unchanged at 7 days post infection; however, as shown in Figure 3, the levels of plasma MPs decreases after 21 days post treatment.

## Discussion

Despite increasing evidence of multidrug-resistant *P. vivax *associated with severe and fatal disease, little is known about its pathogenesis. This study provides the first evidence that microparticles (MPs) from leukocytes, platelets, and erythrocytes are significantly increased in plasma from acute *P. vivax *malaria patients. These results are consistent with the observation that active, non-cytokine, factors are present in paroxysm plasma from non-immune patients with acute *P. vivax *infections [23,24]. As some of the active components in paroxysm plasma are lipids, of unknown nature, and, importantly, are present in particulate form [24], MPs could contribute to *P. vivax *paroxysms. However, at this time, it is not possible to conclude on the role of MPs during paroxysm, because it remains as undefined complex phenomenon, which involves several components, including host-derived cytokines [24].

As virtually all cell types can release MPs, the presence of MPs of different origins circulating during acute *P. vivax *malaria is not unexpected. It rather suggests that different cell types are activated and participate in the myriad of inflammatory processes induced by *P. vivax *parasites/infection. In fact, these data are in concordance with a recently published study reporting that children infected with *P. falciparum *presents high levels of circulating MPs, including those from platelet, erythrocytes, endothelial cells and leukocytes [25]. In these infected Cameroonian's children, MPs levels returned to normal when the patients were cured. This results are in line with the observations described here with *P. vivax*, and by others with *P. falciparum *[17], which shown that the levels of MPs significantly decrease after 19 - 21 days post treatment.

In the present study, elevated levels of platelet-derived microparticles (PMPs) were associated with clinical manifestation of disease, such as fever and prolonged time with malaria symptoms. At this time, the significance of this finding in *P. vivax *infection is not clear, but they are of particular interests as both platelets [26] and PMPs [18] have been shown to play a role in the pathogenesis of malaria caused by *P. falciparum*. Moreover, MPs have been shown to be mediators of the inflammatory process that occur during infection with *Plasmodium berghei *in mice [18]. Recently, Boilard and colleagues [27] provides strong evidence for the pathogenic role of PMPs, confirming their contribution as "incendiary devices" in the conflagration of a hot, swollen, and painful rheumatoid joint.

Although evidence for a role of microparticles in human malaria diseases at present is still only limited, new evidence is accumulating rapidly to support this theory. In Cerebral Malaria, the most severe complications of *P. falciparum *infection, PMPs levels significantly correlated with coma depth and thrombocytopenia [25]. Consequently, these authors have been proposed cell-specific MPs as biological marker for cerebral dysfunctions in severe falciparum malaria, and intervention to block MP production as a new therapeutic target against cerebral malaria.

As in *P. falciparum *PMPs facilitate cytoadherence of iRBCs to brain endothelial [18], it is reasonable to speculate that, similarly, they might be involved in the underlying mechanisms driving pulmonary vascular sequestration in *P. vivax *malaria as suggested by Anstey *et al *[7]. However, as cytoadherence is not a common feature of vivax-infected RBCs and evidences for their microvascular accumulation is, at best, modest [28], it is conceivable that PMPs may be merely an epiphenomenon of the pro-inflammatory response previously observed in vivax malaria [12,29-31]. Further studies will be required to investigate if circulating PMPs from P. vivax patients may act either as biomarker or mediator of inflammation/disease (or both).

A potential limitation of the statistics analysis performed here was that multiple comparison were realized, aiming to find possible associations between MPs and clinical/biological parameters; however, it should be take in consideration that these significant findings were hypothesis-generating, and, consequently, its represent a realistic interpretation of the facts. In addition, multiple linear regression models confirmed the independent association between fever and length of symptoms with platelets-derived microparticles.

An interesting finding was the presence of leukocyte and erythrocyte derived-MPs during acute *P. vivax *malaria. The role of leukocytes in malaria pathogenesis is still not fully understood; nevertheless, it has been shown that these cells are the main source of pro-inflammatory cytokines, especially TNFα which is highly pyrogenic [32]. The presence of MPs derived from these cells provides evidence for their activation in acute vivax malaria. Perhaps, an attractive field of study is to investigate whether or not EMPs might contribute to the distinct erythrocyte destruction commonly observed in vivax malaria [33]. Although severe anemia can be produced by both *P. vivax *and *P. falciparum *infections, malariotherapy studies have shown greater destruction of non-infected RBCs per infected erythrocyte in *P. vivax *as compared to *P. falciparum *malaria [33]. Accordingly, in the present study, the frequency of erythrocyte derived MPs in plasma was not associated with parasitaemia. Actually, the absence of correlation between MPs and parasitaemia is not unexpected, because in the Brazilian Amazon area the wide range of parasitaemia found in *P. vivax *infection does not enable to conclude on the value of this variable as determinant of severity [34].

While it is not possible at this time to characterize the mechanism by which MPs might be involved in the pathogenesis of *P. vivax *malaria, the data shown here raise important questions on the role of cell derived MPs in malaria pathogenesis revealing a fruitful area for investigation. Thus, the potential of circulating cell derived-MPs as non-invasive inflammatory marker of *P. vivax *morbidity is likely.

## Conclusions

This paper describes the initial attempts made at characterization of the MP phenotype in vivax malaria. It is shown here that the plasma levels of platelet, erythrocyte and leukocyte-derived MPs are increased during acute uncomplicated vivax malaria. In *P. vivax *patients platelets derived-MPs were significantly correlated with fever and length of symptoms. Elucidation of the microparticles composition and the mechanisms involved in exertion of their effects could contribute to additional intervention strategies against malaria.

## Competing interests

The authors declare that they have no competing interests.

## Authors' contributions

FFMC and BSF were involved in all stages of this study. CJF coordinated the field study. ATC and CFAB gave substantial constructive advice in the initial design of the project. ALSF and SCOP selected clinically the ovary carcinoma patients. LHC accepts direct responsibility for the conception and coordination of the study. All authors have read and approved the final version of the manuscript.

## Supplementary Material

Additional file 1 **Quantification of platelet, erythrocyte and leukocyte derived-MPs levels in plasma from *P. vivax *patients, before and after antimalarial treatment**. Plasma levels of platelet, erythrocyte and leukocyte derived-MPs were calculated as described in Methods; results are expressed as MPs/μl. The absolute numbers of plasma MPs was compared among healthy donors and *P. vivax *malaria patients. Statistical analysis was performed using the Mann-Whitney two-tailed test. A p value < 0.05 was considered significant. Supplementary Figure. Click here for file
