# Supplementary figures and images for: Augmented plasma microparticles during acute Plasmodium vivax infection
Source: Malar J. 2010 Nov 16;9:327. doi: 10.1186/1475-2875-9-327 (PMC2998527; doi:10.1186/1475-2875-9-327)

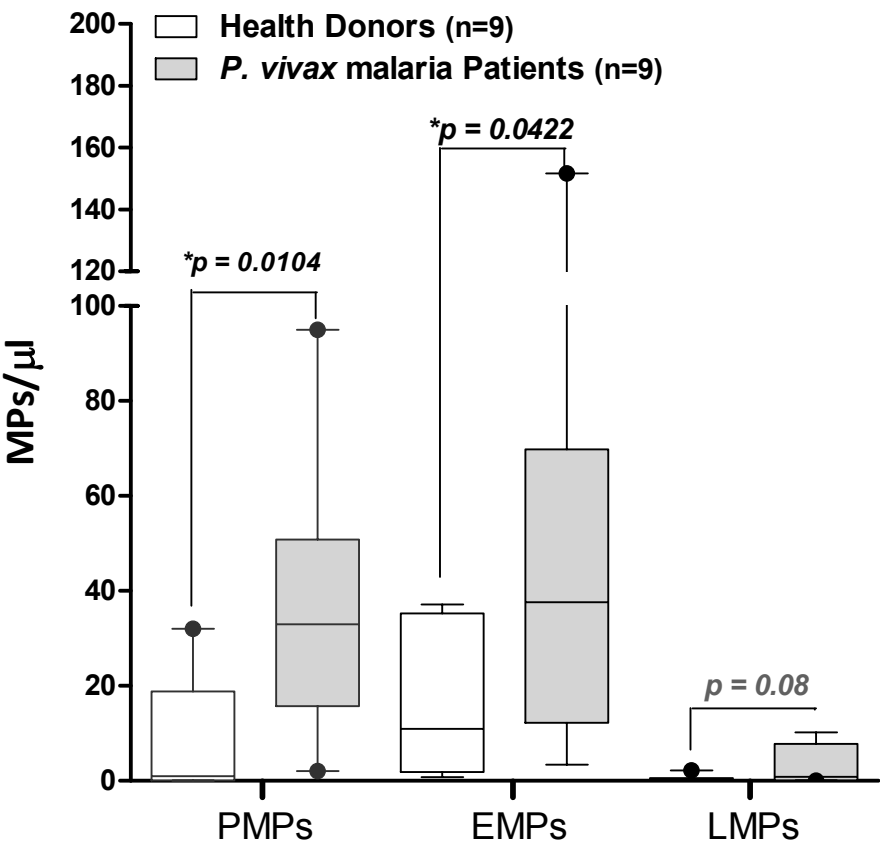

Supplement: Additional file 1 — Quantification of platelet, erythrocyte and leukocyte derived-MPs levels in plasma from P. vivax patients, before and after antimalarial treatment. Plasma levels of platelet, erythrocyte and leukocyte derived-MPs were calculated as described in Methods; results are expressed as MPs/μl. The absolute numbers of plasma MPs was compared among healthy donors and P. vivax malaria patients. Statistical analysis was performed using the Mann-Whitney two-tailed test. A p value < 0.05 was considered significant. Supplementary Figure. [file 1475-2875-9-327-S1.PDF]
